# Routine Clinic Surveillance on Arteriovenous Graft Patency in Hemodialysis Patients with Previous Access Complications

**DOI:** 10.7150/ijms.106651

**Published:** 2025-02-03

**Authors:** Cheng-Chieh Yen, Hung-Pin Tu, Tzu-Chen Lin, Kuan-Ying Li, Szu-Chia Chen

**Affiliations:** 1 Division of Nephrology, Department of Internal Medicine, Ditmanson Medical Foundation Chia-Yi Christian Hospital, Chiayi, Taiwan.; 2 Department of Public Health and Environmental Medicine, School of Medicine, College of Medicine, Kaohsiung Medical University, Taiwan.; 3 Nurse Practitioner, Ditmanson Medical Foundation Chia-Yi Christian Hospital, Chiayi, Taiwan.; 4 Department of Nursing, Chung-Jen Junior College of Nursing, Health Sciences and Management, Taiwan.; 5 Division of Cardiology, Department of Internal Medicine, Ditmanson Medical Foundation Chia-Yi Christian Hospital, Chiayi, Taiwan.; 6 Faculty of Medicine, College of Medicine, Kaohsiung Medical University, Kaohsiung, Taiwan.; 7 Division of Nephrology, Department of Internal Medicine, Kaohsiung Medical University Hospital, Kaohsiung Medical University, Kaohsiung, Taiwan.; 8 Department of Internal Medicine, Kaohsiung Municipal Siaogang Hospital, Kaohsiung Medical University, Kaohsiung, Taiwan.

**Keywords:** arteriovenous graft, outcomes, routine surveillance

## Abstract

**Background:** Arteriovenous grafts (AVGs) are an alternative for hemodialysis (HD) access in patients with inadequate vasculature or advanced age. The effect of routine surveillance for AVG maintenance remains unclear. This study assesses the clinical and economic outcomes of routine surveillance at a collaborative clinic in patients with previous access complications.

**Methods:** We recruited HD patients from the initiation of the clinic in 2020, and divided them into two groups: those receiving routine surveillance and those without. Primary outcomes included AVG interventions (e.g., arteriovenous access [AVA] reconstruction, graft-anastomosis stenting, percutaneous transluminal angioplasty [PTA]). Other outcomes included AVG secondary patency and costs associated with the interventions.

**Results:** Twenty-two patients with routine surveillance and 65 without were recruited. There was no significant difference in AVA reconstruction rate between the surveillance and non-surveillance groups (0.46 *vs.* 0.5 per 100 patient-months, *p* = 0.99), however, rates of graft-anastomosis stenting (0.66 *vs.* 0.2 per 100 patient-months, *p* = 0.02) and PTA (30.19 *vs.* 14.17 per 100 patient-months, *p* < 0.01) were significantly higher in the surveillance group. No significant difference was observed in secondary patency (hazard ratio: 0.83, *p* = 0.79). The total costs of AVG interventions were more than double in the surveillance group (110672 New Taiwan Dollar [NTD] *vs.* 51874 NTD, *p* < 0.01).

**Conclusions:** Routine clinic surveillance in HD patients with AVGs and previous access complications resulted in significantly higher rates of graft-anastomosis stenting, PTA, and associated costs, without significant differences in AVA reconstruction rates or secondary patency. These results highlight the need for further assessment of the cost-effectiveness of routine AVG monitoring.

## Introduction

Hemodialysis (HD) is the primary renal replacement therapy for patients with end-stage renal disease 1. This treatment necessitates the use of a vascular access (VA) such as arteriovenous fistulas (AVFs), arteriovenous grafts (AVGs), and both cuffed and non-cuffed catheters, to effectively remove metabolic waste and excess fluid from the patient's bloodstream. The maintenance of a well-functioning VA is crucial for the optimal management of patients undergoing HD. AVFs are preferred due to their lower thrombosis rates, longer patency, and superior patient outcomes compared to other modalities 2. However, for patients with poor vascular anatomy or advanced age, AVGs may be a suitable alternative 3, despite their significantly lower patency rates compared to AVFs 4-7.

The importance of clinical evaluation of arteriovenous access (AVA) during HD sessions is well-supported by current guidelines 3. The potential benefit of routine surveillance is the ability to preserve AVA patency by identifying dysfunction before full occlusion occurs. Routine surveillance includes monitoring intra-dialysis venous pressure and access flow, allowing for timely preemptive interventions such as percutaneous transluminal angioplasty (PTA), stenting, thrombectomy, bypass surgery, and ultimately access reconstruction. However, studies on AVA surveillance have yielded inconsistent results, with some indicating a beneficial impact on patency 8-10, whereas the outcomes of routine surveillance for AVGs remain ambiguous 11, 12. In addition, the economic impact of these surveillance measures on healthcare systems remains unclear 13-15.

The objective of this study was to assess the impact of routine surveillance at a multidisciplinary clinic in a tertiary teaching hospital on the clinical and economic outcomes of AVGs in HD patients with previous access complications.

## Material and Methods

### Statement of ethical approval

This study was approved by the Institutional Review Board of Ditmanson Medical Foundation Chia-Yi Christian Hospital (approval number: IRB2023067). The need for informed consent was waived due to the retrospective nature of the study. All methods were conducted in compliance with applicable guidelines and regulations 16.

### Data source

To enhance the quality of dialysis VA care, a specialized outpatient clinic was established in our hospital on May 1, 2020, through the collaborative efforts of nephrology and cardiology teams 17. This clinic was designed to address the needs of HD patients with VA complications, such as maturation failure, challenging cannulation, elevated intra-dialysis venous pressure, reduced intra-dialysis blood flow, prolonged post-dialysis hemostasis, unexplained swelling of the limb on the side of the AVA, and other warning signs identified through guideline-directed physical examinations performed by dialysis staff. Nephrologists and dialysis staff actively encourage patients with previous VA complications undergoing interventions to attend routine surveillance at the clinic, which is predominantly led by cardiologists receiving specialized training in vascular access assessment. Following referral, the clinic provides comprehensive and objective access monitoring through physical examinations, supplemented by ultrasound when necessary. Salvage interventions are performed for patients whose VA demonstrates complications or imaging abnormalities, such as inadequate flow to achieve dialysis adequacy or stenosis exceeding 50% as detected by ultrasound. In addition, cardiologists at the clinic engage in periodic discussions with nephrologists and dialysis staff to address complex cases and optimize management plans.

We gathered demographic data including sex, age, HD vintage, and characteristics of AVGs. All AVGs in this study were constructed using expanded polytetrafluoroethylene grafts. We also collected information on comorbidities, history of parathyroidectomy, use of far-infrared radiation therapy, administration of antiplatelet and antihypotensive agents, and laboratory data at the time of recruitment. Furthermore, data on episodes and costs associated with AVG interventions, including AVA reconstruction, graft-anastomosis stenting, PTA, ultrasound for the AVG interventions and clinic visits were extracted from the hospital's electronic medical records system. The datasets generated or analyzed in this study are available from the corresponding author upon reasonable request.

### Study design

**Figure 1:**
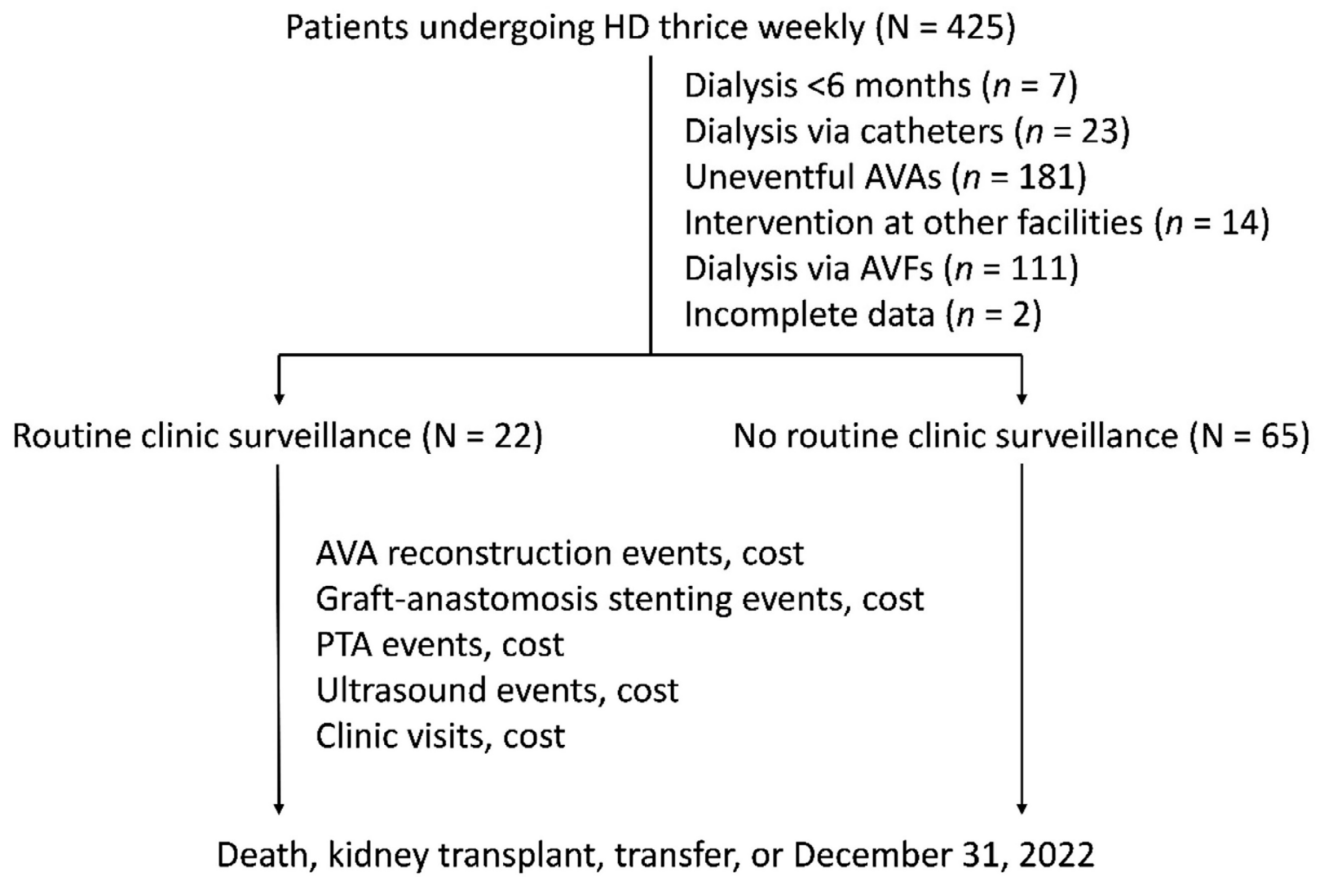
Flow diagram of the recruitment, allocation, and follow-up. AVA: arteriovenous access; AVF: arteriovenous fistula; HD: hemodialysis; PTA: percutaneous transluminal angioplasty.

We recruited patients undergoing HD thrice weekly from the clinic's launch date. The exclusion criteria were patients undergoing dialysis for less than 6 months (*n* = 7), those using catheters or AVFs (*n* = 134) for dialysis, those who had not undergone salvage interventions for their AVAs (*n* = 181), those receiving interventions at other facilities (*n* = 14), and those with incomplete data (*n* = 2). The recruited patients were then categorized into two groups: those receiving routine clinic surveillance and those without. Routine clinic surveillance was defined as regular clinic visits scheduled at intervals ranging from 3 to 6 months, commencing from the establishment date of the clinic. Patients in the routine clinic surveillance group who experienced VA complications outside of the scheduled visit intervals could arrange additional appointments as needed. The follow-up period ended at the patient's death, kidney transplant, transfer to another medical facility, or December 31, 2022, whichever occurred first. Figure 1 depicts the recruitment, allocation, and follow-up process. In total, the study analyzed 87 patients, with 22 (25.3%) receiving routine clinic surveillance and 65 (74.7%) not receiving routine clinic surveillance.

The primary outcomes of this study were the rates of different AVG interventions among the recruited patients throughout the follow-up period. AVA reconstruction was defined as the creation of a new VA after the previous one failed to support adequate dialysis treatment. Sensitivity analysis was performed to validate our findings. We also compared the secondary patency of AVGs between groups, defined as the duration from recruitment to AVG abandonment, and analyzed the costs associated with AVG interventions. Furthermore, we evaluated correlations between patient demographic and AVA reconstruction.

### Statistical analysis

Statistical analyses were conducted using MedCalc Statistical Software (version 23.0.6, MedCalc Software Ltd., Ostend, Belgium). Categorical variables were expressed as frequencies or percentages, while continuous variables were presented as means and standard deviations. The chi-square test or Mann-Whitney test was used for comparisons between variables, as appropriate. A Cox proportional hazards regression model was used to assess the impact of routine clinic follow-up on time to AVA reconstruction. The model estimated hazard ratios after adjusting for potential confounders and significant variables between groups, to provide robust estimates of the association between routine follow-up and the risk of AVA reconstruction. Logistic regression analysis was used to determine the crude odds ratios between categorical variables and outcomes, while Spearman's rank correlation coefficients were calculated to assess correlations between continuous variables and outcomes. Statistical significance was set at a two-tailed *p* value of less than 0.05.

## Results

The baseline demographic and clinical characteristics of the recruited patients are presented in Table 1. No significant difference was observed in sex distribution between the two groups, and there were also no significant differences in age, HD vintage, AVG characteristics, comorbidities, history of parathyroidectomy, use of far-infrared radiation therapy, or administration of antiplatelet or antihypotensive agents. Regarding laboratory data, the patients undergoing routine clinic surveillance had a significantly higher hemoglobin level compared to those not receiving routine follow-up (10.8 g/dL *vs.* 10.1 g/dL, *p* < 0.01). Other laboratory parameters were comparable between the two groups.

The primary outcomes of the patients with and without routine clinic surveillance are detailed in Table 2. The rates of AVG interventions were expressed as occurrence per 100 patient-months, adjusted for HD vintage. No significant difference in the rate of AVA reconstruction was observed between the patients with and without routine clinic surveillance (0.46 / 100 patient-months *vs.* 0.5 / 100 patient-months, *p* = 0.99). Conversely, the rates of graft-anastomosis stenting (0.66 / 100 patient-months *vs.* 0.2 / 100 patient-months, *p* = 0.02) and PTA (30.19 / 100 patient-months *vs.* 14.17 / 100 patient-months, *p* < 0.01) were significantly higher in the patients with routine clinic surveillance. Sensitivity analysis, including patients matched for hemoglobin level, patients aged over 65 years, and those receiving antiplatelet agents, corroborated our primary findings.

**Figure 2:**
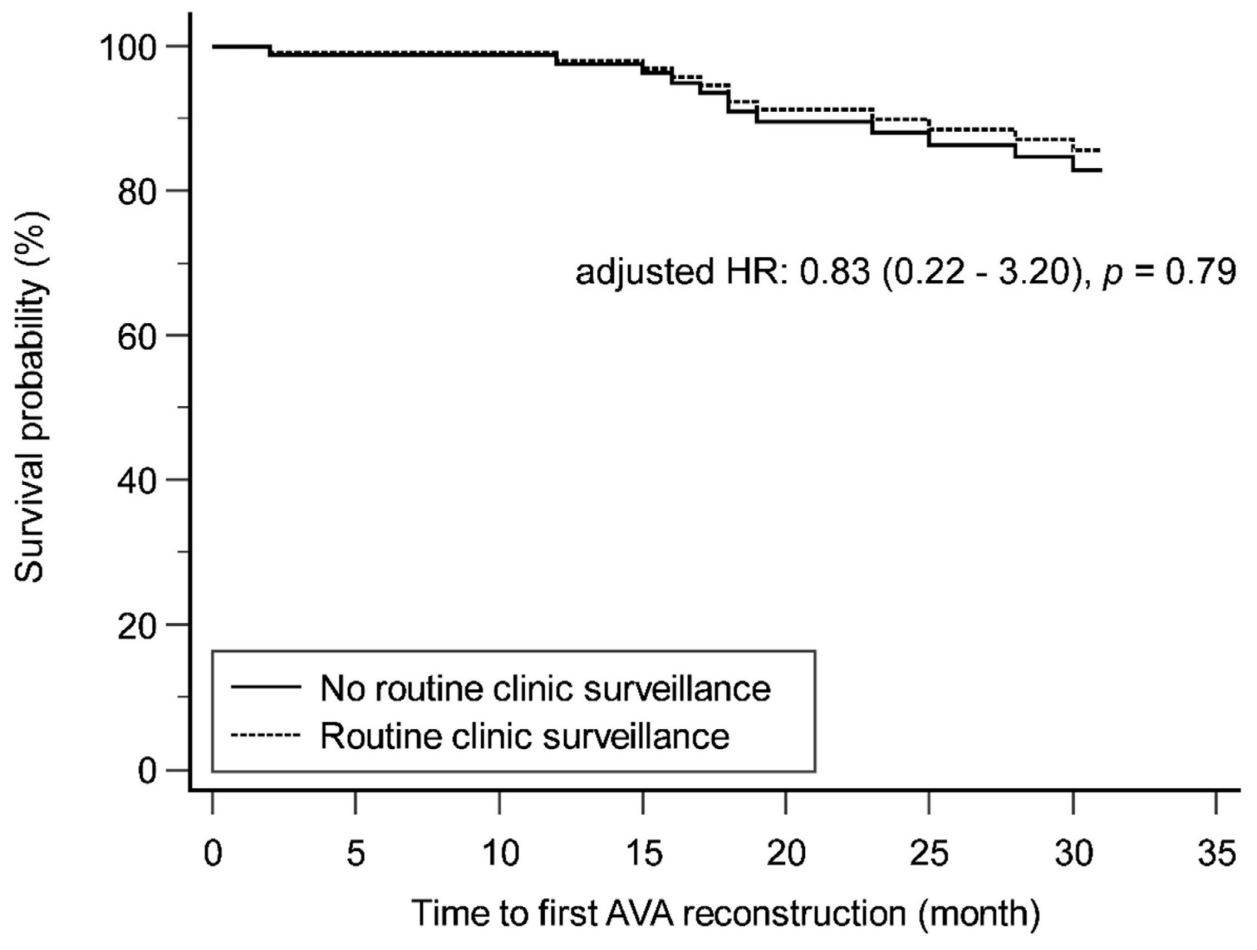
Secondary patency of AVG in patients with and without routine clinic surveillance. AVA: arteriovenous access; AVG: arteriovenous graft; HR: hazard ratio.

The secondary patency of AVGs between the patients with and without routine clinic surveillance is depicted in Figure 2, using the Cox proportional hazards regression model. After adjusting for hemoglobin level, the survival probability did not differ significantly between the two groups (adjusted hazard ratio: 0.83, *p* = 0.79).

The costs of AVG interventions expressed in New Taiwan Dollars (NTD) per patient during the follow-up period in both groups are detailed in Table 3. The cost of AVA reconstruction was lower in the patients with routine clinic surveillance compared to those without, although the difference was not significant (9414 NTD per patient *vs.* 10620 NTD per patient, *p* = 0.96). However, the costs of graft-anastomosis stenting (2563 NTD per patient *vs.* 434 NTD per patient, *p* = 0.02) and PTA (91309 NTDs per patient *vs.* 38548 NTD per patient, *p* < 0.01) were significantly higher in the patients with routine clinic surveillance. Overall, the total costs of AVG interventions, including the costs of ultrasound and clinic visits (shown in Table 4), were significantly higher in the routine surveillance group compared to the group without routine surveillance (110672 NTD per patient *vs.* 51874 NTD per patient, *p* < 0.01).

The correlations between patient demographic information and AVA reconstruction are presented in Supplementary Table 1 and 2. Analysis revealed no significant correlations between categorical variables Supplementary Table 1 or continuous variables Supplementary Table 2 and AVA reconstruction in the patients with routine clinic surveillance.

## Discussion

This study investigated the outcomes of routine clinic surveillance on AVG patency in HD patients with previous access complications. The results showed no significant differences in AVA reconstruction or secondary patency rates, but significant increases in graft-anastomosis stenting and PTA rates in the patients undergoing routine clinic surveillance compared to those without. Furthermore, the patients with routine clinic surveillance incurred significantly higher costs to maintain AVG patency. These findings underscore the need for more detailed evaluations of the cost-effectiveness of routine AVG monitoring in this patient cohort.

AVA reconstruction is typically required when an access fails to maintain adequate function despite salvage interventions 3, and routine surveillance may help reduce its occurrence. In this study, we observed no significant difference in AVA reconstruction rate between the patients undergoing routine clinic surveillance and those without, a finding validated by sensitivity analysis involving matched hemoglobin level, as well as subgroups of older patients and those on antiplatelet therapy. McCarley *et al.* evaluated the clinical and financial outcomes of different VA surveillance methods in a cohort of 132 HD patients 18. Their results showed variable costs in the patients with AVGs, reflecting differences in the rates of access reconstruction or revision across surveillance methods, compared to a control group without surveillance 18. The discrepancies between our findings and those of McCarley *et al.* may be attributed to differences in the definition of AVA reconstruction.

Several salvage interventions are available to restore the patency of AVGs, with graft-anastomosis stenting and PTA being the primary approaches in our hospital. Graft-anastomosis stenting is indicated for cases requiring frequent PTA at the stenotic graft-anastomosis site. In our study, both graft-anastomosis stenting and PTA rates were significantly higher in the routine clinic surveillance group, a finding that was further supported by sensitivity analysis. Moist *et al.* conducted a randomized controlled trial (RCT) involving 112 HD patients to assess the effect of monthly AVG flow monitoring on thrombosis and access loss, and found that the intervention rates in the treatment group were 1.65 times higher than those in the control group 19. Similarly, a prospective RCT by Robbin *et al.* assessed the impact of regular ultrasound surveillance on stenosis in 126 HD patients with AVGs, and found that the frequency of preemptive PTA was 64% higher in the ultrasound surveillance group compared to the control group 20. In addition, Hoeben *et al.* assessed the impact of routine surveillance on intervention rates in a cohort of 86 patients, and observed that the frequency of interventions was significantly higher in the group receiving regular surveillance compared to those without 21. Moreover, in a cohort of 363 patients, Plantinga *et al.* observed that those undergoing more frequent monitoring were 1.4 times more likely to require an intervention compared to those with less frequent monitoring 22. Furthermore, national data from the Netherlands reported by Tordoir *et al.* indicated that multidisciplinary discussions of AVA problems increased the rate of preemptive endovascular interventions 23. In a cohort of 60 patients, Mauro *et al.* assessed AVG secondary patency between patients in a surveillance program and those receiving clinical assessment 24. In the surveillance group, 15 AVG malfunctions were detected and treated with graft-anastomosis stenting and PTA, while no malfunctions were observed in the historical control group 24. Despite these findings, some studies have reported minimal differences in intervention rates. The Hemodialysis Access Surveillance Evaluation Study, a multicenter RCT by Salman *et al.* compared monthly ultrasound AVA flow surveillance with standard care in 436 HD patients, and found no statistically significant difference in the total number of procedures between the groups 25. Similarly, Schuman *et al.* compared AVA outcomes between ultrasound-based flow measurements and clinical criteria, and reported a modest 1.17-fold increase in intervention rate in the ultrasound group only 26. These discrepancies may be due to differences in study populations and design.

Maintaining AVA functionality is a crucial issue in HD-related research. In our study, secondary patency of AVGs was defined as the period from the initiation of the clinic to the date of AVG abandonment. Cox proportional hazards regression was used to compare secondary patency between the patients with and without routine clinic surveillance. After adjusting for baseline demographic and clinical variables, no significant difference in AVG secondary patency was identified between the two groups. Ram *et al.* conducted an RCT involving 101 patients, and applied criteria including clinical symptoms, AVG flow measurements, and ultrasound findings to guide referrals for PTA, and they observed no significant difference in 2-year AVG survival rate between the groups 27. Similarly, Dember *et al.* performed an RCT of 64 patients to compare the prophylactic repair of AVG stenosis with repair at the time of thrombosis 28. Over the 3.5-year study period, no significant differences were observed in AVG abandonment rates or time to abandonment between the intervention and observation groups 28. These findings are consistent with other RCTs 19, 20. In addition, Lumsden *et al.* compared a surveillance program involving prophylactic PTA for stenoses greater than 50% with a non-interventional approach in 65 patients, and found no significant differences in patency rates at 6 and 12 months between the groups 29. These outcomes align with similar findings from other cohort studies 22, 24. In contrast, Mauro *et al.* compared AVG secondary patency between patients in a surveillance program and those undergoing clinical assessment, and found that the 5-year patency rate was significantly higher in the surveillance group 24. However, it is important to acknowledge that the comparison groups were analyzed in different temporal and geographic contexts. Overall, our findings align with the existing literature, including subgroup analysis in systematic reviews and meta-analyses by Tonelli *et al.* 30 and Casey *et al.* 8 which found no significant difference in AVG abandonment when comparing AVA flow surveillance with standard care.

The economic burden of HD places a significant strain on healthcare systems 31, and the costs associated with AVA interventions further exacerbate this challenge 32. The costs related to graft-anastomosis stenting and PTA were significantly higher in the routine surveillance group compared to those without surveillance in this study. Consequently, the total costs of AVG-related interventions were more than twice as high in the routine surveillance group compared to the patients without routine surveillance. In the RCT by Ram *et al.* mentioned above, subgroup analysis revealed that costs related to monthly AVG flow monitoring, quarterly stenosis evaluations, and total PTAs were higher in the surveillance group 13. These findings align with the cost outcomes observed in our study. In contrast, McCarley *et al.* reported a 49% reduction in the total costs of managing thrombosis-related events in AVGs with ultrasound-assisted flow monitoring compared to no monitoring, and a 54% reduction compared to venous pressure monitoring 18. Given that AVG interventions were primarily managed on an outpatient basis and the demographic and clinical characteristics were comparable between the surveillance and non-surveillance groups, our study specifically examined the costs associated with outpatient interventions, excluding expenses associated with dialysis catheters and hospitalizations. Further investigations are warranted to evaluate the clinical benefits and cost-effectiveness of routine surveillance, including an analysis of potential long-term benefits, such as reduced hospitalizations and complication rates.

The Kidney Disease Outcomes Quality Initiative guidelines recommend regular physical examinations of AVGs by experienced practitioners to identify clinical signs of flow dysfunction. However, routine surveillance methods, including AVG flow measurement, pressure monitoring, or imaging for stenosis beyond standard clinical monitoring, are not advised for improving AVG patency 3. We have established a multidisciplinary clinic with bidirectional feedback, involving nephrologists, dialysis staff, and trained cardiologists, in conjunction with guideline-directed assessments in the dialysis unit, to optimize AVA patency in our HD patients. This collaborative approach offers a fresh perspective on AVG follow-up. However, our surveillance strategy did not significantly improve AVG secondary patency and was associated with higher intervention rates and increased costs of AVG-related care.

This study also has several limitations. First, being a retrospective analysis from a single tertiary teaching hospital, the findings may not be widely generalizable. Future research involving larger sample sizes or multicenter data is recommended to validate these results and improve their applicability. Additionally, the follow-up period for the recruited patients was limited to a maximum of 1.5 years, and longer follow-up durations may be necessary to fully assess long-term outcomes. Second, the collaborative clinic adopted an individualized approach rather than a standardized protocol for the assessment and management of AVG. Furthermore, the multidisciplinary team lacked the inclusion of vascular surgeons, who play a critical role in AVA reconstruction. Third, critical variables such as AVG flow, intra-dialysis venous pressure, and outcomes such as dialysis catheter use and associated hospitalization data were not recorded. Fourth, the reasons for AVA reconstruction are not limited to AVG occlusion 28, however they were not specified in our analysis. Lastly, despite multivariate analysis was performed to account for known variables, the influence of unmeasured confounding factors, such as smoking status, on the outcomes cannot be completely excluded.

## Conclusions

We observed no significant differences in AVA reconstruction or secondary patency rate between HD patients with AVGs receiving routine collaborative clinic surveillance and those without. However, routine clinic surveillance was associated with a marked increase in graft-anastomosis stenting and PTA, resulting in significantly higher costs for maintaining AVG patency. These findings highlight the need for further assessment of the cost-effectiveness of routine AVG surveillance in this patient population.

## Supplementary Material

Supplementary tables.

## Figures and Tables

**Table 1:** Baseline demographic and clinical characteristics of patients with and without routine clinic surveillance

Parameters	Routine surveillance (N=22)	No routine surveillance (N=65)	*p*
Sex (male / female)	9 / 13	29 / 36	0.76
Age (years)	75 ± 14	72 ± 10	0.10
HD vintage (months)	108 ± 72	112 ± 109	0.51
AVG at left / right side	19 / 3	57 / 8	0.87
AVG at forearm / arm	14 / 8	44 / 21	0.73
Hypertension	19	59	0.56
DM	9	37	0.20
Heart failure	3	10	0.84
Cardiovascular disease	7	30	0.24
Cerebrovascular disease	8	12	0.09
Peptic ulcer disease	16	45	0.76
Gout / hyperuricemia	11	25	0.35
Cancer	4	18	0.38
HBV carrier	1	7	0.39
HCV carrier	4	15	0.63
Parathyroidectomy	6	7	0.06
FIR therapy	3	4	0.27
Antiplatelet agents	11	32	0.95
Antihypotensive agents	5	11	0.55
Leukocyte (10^3^/μL)	7.02 ± 2.20	6.66 ± 2.14	0.54
Hemoglobin (g/dL)	10.8 ± 1.2	10.1 ± 0.9	<0.01
Platelet (10^3^/μL)	169 ± 47	195 ± 64	0.13
Glucose (mg/dL)	151 ± 52	151 ± 76	0.34
HbA1c (%, DM patients)	6.5 ± 1.4	6.9 ± 2.0	0.74
Albumin (g/dL)	3.9 ± 0.3	3.9 ± 0.3	0.75
ALK-P (U/L)	274 ± 124	329 ± 180	0.16
Blood urea nitrogen (mg/dL)	69 ± 17	73 ± 20	0.42
Creatinine (mg/dL)	9.9 ± 3.1	9.7 ± 2.4	0.98
Potassium (mmol/L)	4.3 ± 0.7	4.7 ± 0.7	0.05
Phosphorus (mg/dL)	4.8 ± 1.6	5.0 ± 1.6	0.63
Total calcium (mg/dL)	9.2 ± 0.7	9.0 ± 0.8	0.08
Sodium (mmol/L)	139 ± 4	138 ± 4	0.43
Kt/V	1.7 ± 0.3	1.6 ± 0.2	0.20
Urea reduction ratio (%)	76 ± 4	74 ± 5	0.15
Uric Acid (mg/dL)	7.2 ± 1.9	7.1 ± 2.1	0.86
Cholesterol (mg/dL)	165 ± 31	158 ± 38	0.29
Triglyceride (mg/dL)	177 ± 124	158 ± 109	0.58
HDL-C (mg/dL)	43 ± 13	45 ± 16	0.69
LDL-C (mg/dL)	94 ± 28	88 ± 32	0.27
Serum iron (μg/dL)	73 ± 22	68 ± 31	0.23
TIBC (μg/dL)	226 ± 36	231 ± 55	0.93
Transferrin saturation (%)	33 ± 10	30 ± 13	0.22
Ferritin (ng/mL)	558 ± 273	504 ± 356	0.26
PTH-I (pg/mL)	327 ± 266	319 ± 368	0.44
hs-CRP (mg/dL)	0.86 ± 1.09	1.74 ± 2.80	0.30

Abbreviations: ALK-P: alkaline phosphatase; AVG: arteriovenous graft; DM: diabetes mellitus; FIR: far-infrared radiation; HbA1c: glycated hemoglobin; HBV: hepatitis B virus; HCV: hepatitis C virus; HD: hemodialysis; HDL-C: high density lipoprotein cholesterol; hs-CRP: high sensitivity C-reactive protein; LDL-C: low density lipoprotein cholesterol; PTH-I: parathyroid hormone intact; TIBC: total iron-binding capacity.

**Table 2:** AVG interventions rates and associated sensitivity analyses in patients with and without routine clinic surveillance

Parameters	Routine surveillance	No routine surveillance	*p*
	N = 22	N = 65	
AVA reconstruction rate (per 100 patient-months)	0.46 ± 1.19	0.50 ± 1.30	0.99
Graft-anastomosis stenting rate (per 100 patient-months)	0.66 ± 1.45	0.20 ± 1.30	0.02
PTA rate (per 100 patient-months)	30.19 ± 13.68	14.17 ± 10.43	<0.01
Sensitivity analyses			
*Matched with hemoglobin*	N = 22	N = 22	
AVA reconstruction rate	0.46 ± 1.12	0.73 ± 1.70	0.68
Graft-anastomosis stenting rate	0.66 ± 1.45	0.00 ± 0.00	0.04
PTA rate	30.19 ± 13.68	13.81 ± 8.25	<0.01
*Aged over 65 years*	N = 19	N = 52	
AVA reconstruction rate	0.36 ± 1.10	0.62 ± 1.43	0.53
Graft-anastomosis stenting rate	0.76 ± 1.53	0.19 ± 1.39	<0.01
PTA rate	31.56 ± 14.26	14.62 ± 11.14	<0.01
*Receiving antiplatelet agents*	N = 11	N = 32	
AVA reconstruction rate	0.00 ± 0.00	0.30 ± 0.96	0.30
Graft-anastomosis stenting rate	0.29 ± 0.97	0.00 ± 0.00	0.09
PTA rate	28.39 ± 9.48	14.78 ± 12.75	<0.01

Abbreviations: AVA: arteriovenous access; AVG: arteriovenous graft; PTA: percutaneous transluminal angioplasty.

**Table 3:** AVG interventions costs in patients with and without routine clinic surveillance

Parameters	Routine surveillance (N=22)	No routine surveillance (N=65)	*p*
AVA reconstruction costs (NTD per person)	9414 ± 24248	10621 ± 27911	0.96
Graft-anastomosis stenting costs (NTD per person)	2563 ± 5566	434 ± 2454	0.02
PTA costs (NTD per person)	91309 ± 41858	38548 ± 30783	<0.01
Total costs (NTD per person)	110672 ± 62064	51874 ± 48400	<0.01

Abbreviations: AVA: arteriovenous access; AVG: arteriovenous graft; NTD: New Taiwan Dollar; PTA: percutaneous transluminal angioplasty.

**Table 4:** Ultrasound and clinic visits in patients with and without routine clinic surveillance

Parameters	Routine surveillance (N=22)	No routine surveillance (N=65)	*p*
Ultrasound rate (per 100 patient-months)	15.07 ± 12.64	3.36 ± 4.80	<0.01
Clinic rate (per 100 patient-months)	50.10 ± 21.63	21.20 ± 17.19	<0.01
Ultrasound costs (NTD per patient)	3382 ± 2910	714 ± 960	<0.01
Clinic costs (NTD per patient)	4004 ± 1718	1558 ± 1349	<0.01

Abbreviations: NTD: New Taiwan Dollar
