# Supplementary material for: Routine Clinic Surveillance on Arteriovenous Graft Patency in Hemodialysis Patients with Previous Access Complications
Source: Int J Med Sci. 2025 Feb 3;22(5):1064–71. doi: 10.7150/ijms.106651 (PMC11866542; doi:10.7150/ijms.106651)
Supplement: Supplementary file 1 — Supplementary tables. [file ijmsv22p1064s1.pdf]

Supplementary Material

Supplementary Table 1. Regression analyses of AVA reconstruction in patients with routine clinic surveillance

Parameters	Crude odds ratio	*p*
Sex	0.69	0.77
AVG at left / right side	0.24	0.31
AVG at forearm / arm	1.17	0.91
Hypertension	0.24	0.31
DM	0.69	0.77
Heart failure	4.25	0.31
Cardiovascular disease	<0.01	1.00
Cerebrovascular disease	<0.01	1.00
Peptic ulcer disease	0.71	0.80
Gout / hyperuricemia	<0.01	1.00
Cancer	<0.01	1.00
HBV carrier	0.00	1.00
HCV carrier	2.67	0.48
Parathyroidectomy	7.50	0.13
FIR therapy	0.00	1.00
Antiplatelet agents	<0.01	1.00
Antihypotensive agents	1.88	0.64

Abbreviations: AVA: arteriovenous access; AVG: arteriovenous graft; DM: diabetes mellitus; FIR: far-infrared radiation; HBV: hepatitis B virus; HCV: hepatitis C virus.

Supplementary Table 2. Correlation analyses of AVA reconstruction in patients with routine clinic surveillance

Parameters	Correlation coefficient	*p*
Age	-0.12	0.60
HD vintage	0.02	0.92
Leukocyte	-0.01	0.95
Hemoglobin	-0.08	0.71
Platelet	0.01	0.95
Glucose	-0.05	0.82
HbA1c (in DM patients)	0.41	0.27
Albumin	0.06	0.79
ALK-P	0.06	0.79
Blood urea nitrogen	0.13	0.58
Creatinine	0.03	0.88
Potassium	0.04	0.85
Phosphorus	0.01	0.96
Total calcium	-0.13	0.56
Sodium	-0.04	0.86
Kt/V	0.14	0.52
Urea reduction ratio	0.10	0.65
Uric Acid	0.13	0.55
Cholesterol	0.19	0.40
Triglyceride	0.08	0.74
HDL-C	<-0.01	0.99
LDL-C	0.27	0.23
Serum iron	-0.03	0.91
TIBC	-0.16	0.49
Transferrin saturation	0.06	0.80
Ferritin	0.14	0.54
PTH-I	0.31	0.17
hs-CRP	-0.19	0.41

Abbreviations: ALK-P: alkaline phosphatase; AVA: arteriovenous access; DM: diabetes mellitus; HbA1c: glycated hemoglobin; HD: hemodialysis; HDL-C: high density lipoprotein cholesterol; hs-CRP: high sensitivity C-reactive protein; LDL-C: low density lipoprotein cholesterol; PTH-I: parathyroid hormone intact; TIBC: total iron-binding

capacity
